# Age and pupil size: key predictors of mortality in traumatic brain injury patients with GCS 3

**DOI:** 10.3389/fneur.2025.1536421

**Published:** 2025-04-04

**Authors:** Tee-Tau Eric Nyam, Kuan-Chi Tu, Yun-Hsuan Kuo, Che-Chuan Wang, Chung-Feng Liu, Jen-Chieh Liao, Ching-Lung Kuo

**Affiliations:** ^1^Department of Neurosurgery, Chi Mei Medical Center, Tainan, Taiwan; ^2^Department of Clinical Psychology, Chung Shan Medical University, Taichung, Taiwan; ^3^Department of Medical Research, Chi Mei Medical Center, Tainan, Taiwan; ^4^School of Medicine, College of Medicine, National Sun Yat-Sen University, Kaohsiung, Taiwan

**Keywords:** traumatic brain injury, mortality, emergency room triage, GCS 3, pupillary reaction, pupil size

## Abstract

This study investigates the relationship between mortality and specific clinical factors in patients with severe traumatic brain injury (TBI) who present with a Glasgow Coma Scale (GCS) score of 3. Data from 161 adult patients were collected from the Chi-Mei Medical Center in Taiwan, spanning 2010 to 2019. The findings revealed an overall mortality rate of 44.10%, with significant predictors of mortality identified as age and pupil size. The Spearman correlation analysis showed that both age and pupil sizes were positively correlated with mortality rates. Multiple logistic regression confirmed age and left pupil size as strong predictors of mortality. Patients with GCS 3 and both unreactive pupils measuring 4 mm or more experienced the highest mortality rate of 68.39%, while those with pupils less than 4 mm had a lower mortality rate of 32.26%. The study determined optimal cut-off values for age and pupil size using ROC and AUC analysis, highlighting the significance of age in mortality predictions. These findings underscore the critical role of age and pupil size in the prognosis of TBI patients and provide valuable guidance for clinicians managing such cases.

## Introduction

Traumatic brain injury (TBI) rates have risen steadily in the US, from 521 cases per 100,000 in 2001 to 824 in 2010 (1). After TBI, mortality rates are about 11.2 per 100,000 in Europe (57,000 deaths) and 17.7 per 100,000 in the US (53,000 deaths) (2, 3). In Taiwan, TBI mortality rates range from 26.15 to 36.36% between 1997 and 2007 (4, 5). TBI remains a significant public health concern, straining resources and impacting patients’ well-being (6).

The Glasgow Coma Scale (GCS), introduced by Teasdale and Jennett (7) and revised in 1976, assesses eye, verbal, and motor responses, with scores ranging from 3 to 15. A score of 8 or lower indicates critical condition requiring intubation (7, 8). A GCS score of 3 at initial assessment is strongly linked to poor prognosis (9–13), with mortality rates between 49.2% (12) and 89% (9). Physicians facing TBI patients with a GCS score of 3 grapple with questions regarding survival, aggressive treatments, and potential recovery.

Several studies have identified key predictive factors for TBI outcomes, including age and surgery (14), sex (15), obesity (16), GCS, pupil reactivity (17), midline shift in computed tomography (CT) findings (18), coagulopathy (19) and associated extracranial injuries (20). Age, GCS, pupil reactivity, brain CT findings, and intracranial pressure are particularly important for patients with severe initial assessments. However, most predictors require post-admission evaluation through blood sampling, imaging studies, or surgery. Early mortality risk prediction and detailed pupil size assessment in ER triage remain areas requiring further clarification.

Recently, our team has developed a computer-assisted system that eliminates the need for brain CT scans and blood sampling to predict early mortality risk in TBI patients during the emergency room triage process (21). This predictive model incorporates essential twelve variables (22, 23). These variables are recognized as significant prognostic indicators for trauma patients. This study aimed to predict mortality risk for TBI patients with a GCS score of 3 at ER arrival using twelve variables. We analyzed the hospital’s TBI database retrospectively to identify key factors associated with early outcomes, aiming to inform healthcare decisions and educate patient families.

## Materials and methods

### Patient selection

**Figure 1:**
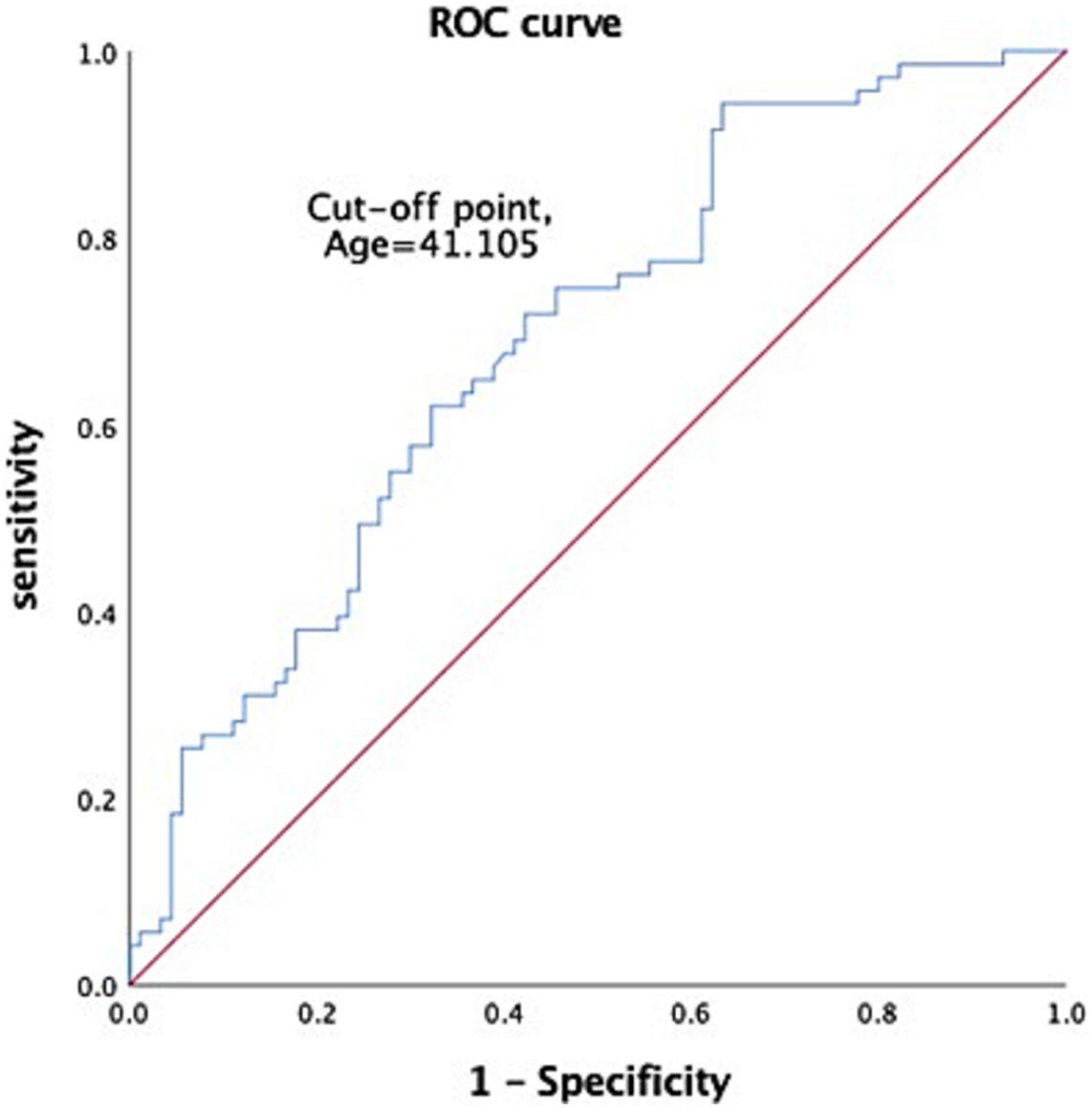
Study flow diagram.

All head injury patients who presented at the emergency room triage of Chi-Mei Medical Center between 2010 and 2019 were retrospectively included in this study. The inclusion criteria were as follows: patients with head trauma who were admitted to the ER and had the following diagnostic codes—ICD-9: 800*-804*, 850*-854*, 959.0, 959.01, 959.8–959.9, ICD-10: S00*-T07*. Patients with missing or ambiguous values were excluded. Other patients and the public were not involved in any way in this research. Figure 1 displays the study flow diagram.

The twelve feature variables including patients’ age, gender, body mass index (BMI), Taiwan Triage and Acuity Scale (TTAS), heart rate, body temperature, respiratory rate, GCS, left and right pupil size, and light reflex were enrolled due to their wide availability in the triage setting. Dilated pupils were defined as ≥4 mm in diameter (11). The presence of unresponsive pupil is defined as unresponsive (<1 mm) to a light stimulus (19, 24). The pupillary light reflex was divided into three categories including both unreactive, one unreactive and both reactive. All patients in our hospital received the standard management protocol for TBI patients. Specifically, following surgery, patients underwent cerebral perfusion-guided management with the goal of maintaining a cerebral perfusion pressure of 60 mmHg or higher and ensuring intracranial pressure remained at 20 mmHg or lower. The recorded outcome was categorized as either mortality or survival.

### Statistical analysis

Significant testing was conducted using the t-test for numerical variables and the Chi-square test for categorical variables. Additionally, data were analyzed using Spearman’s correlation method to demonstrate the strength of the correlation between each feature and mortality. Multiple logistic regression models were used for correlation between two variables and defined the independent risk factors for poor outcome. Using the Receiver Operating Characteristic (ROC) and the area under curve (AUC), variables cutoff value and its reliability in prognosis could be estimated (25). Commercial statistical software (SPSS for Windows, Version 15, SPSS Inc., Chicago, IL, United States) was utilized for this analysis. *p*-values less than 0.05 were considered statistically significant.

### Ethics

This study obtained ethics approval (IRB no. 10911-006) from the Institutional Review Board of Chi Mei Medical Center in Tainan, Taiwan. The authors conducted all methods in compliance with applicable guidelines and regulations. The Ethics Committee waived the need for informed consent due to the retrospective nature of the study.

## Results

A total of 161 patients were retrospectively included from the electronic medical records system of Chi-Mei Hospital. There were 118 males and 43 females. The average age was 55.64 ± 17.41 years (mean ± SD). Seventy-one patients died, and the total mortality rate was 44.10% (71/161). The survival time from ER arrival to discharge is 6.18 ± 5.68 days. Among these 11 features, age (*p* < 0.001**), left and right pupil size (*p* < 0.001**), and pupil light reflex (*p* = 0.001**) showed a significant difference in relation to mortality. Table 1 provides detailed information about the demographics and clinical pictures of patients with TBI.

**Table 1:** Demographics and clinical pictures in patients with traumatic brain injury.

Variable	Overall *N* = 161	Mortality *N* = 71	Survival *N* = 90	*p*
Age, M (SD)	55.64 (17.41)	62.32 (14.75)	50.37 (17.61)	<0.001**
BMI, M (SD)	24.29 (4.27)	24.07 (3.62)	24.47 (4.73)	0.563
Heart rate, M (SD)	88.12 (33.65)	82.52 (14.70)	92.53 (24.96)	0.061
Body temperature M (SD)	36.09 (0.58)	36.10 (0.63)	36.09 (0.54)	0.904
Respiratory rate	16.39 (6.41)	15.54 (7.95)	17.07 (4.80)	0.133
Sex, *n* (%)				
Male	118 (73.3)	54 (76.1)	64 (28.9)	0.496
Female	43 (26.7)	17 (23.9)	26 (71.1)	
TTAS, *n* (%)				
Level I	153 (95)	69 (97.2)	84 (93.3)	1.535
Level II	7 (4.3)	2 (2.8)	5 (5.6)	
Level III	1 (0.6)	0 (0)	1 (1.1)	
Pupil light reflex, *n* (%)				
Both reactive	52 (32.3)	12 (16.9)	40 (4.44)	0.001**
One unreactive	14 (8.7)	6 (8.5)	8 (8.89)	
Both unreactive	95 (59)	53 (74.6)	42 (46.67)	
Pupil size (L), M (SD)	3.34 (1.41)	3.90 (1.44)	2.89 (1.22)	<0.001**
Pupil size (R), M (SD)	3.33 (1.46)	3.88 (1.50)	2.89 (1.27)	<0.001**

A *t*-test was used for numerical variables and the Chi-square test was used for categorical variables; BMI: body mass index; TTAS: Taiwan Triage and Acuity Scale. * *p* < 0.05; ** *p* < 0.005.

Table 2 displays the correlations between various features and mortality, measured using the Spearman correlation coefficient (r). Among these features, age (*r* = 0.342, *p* < 0.001**) and the size of the left (*r* = 0.357, *p* < 0.001**) and right pupils (*r* = 0.357, *p* < 0.001**), as well as both unreactive pupil light reflex (*r* = 0.282, *p* < 0.001**), exhibit significant positive correlations with mortality. This underscores their substantial impact on prediction.

**Table 2:** The Spearman correlation coefficient for each feature and mortality.

Features	*r*	*p*
Age	0.342	<0.001**
Sex		
Male	0.55	0.484
Female	−0.055	0.484
TTAS	−0.096	0.223
BMI	−0.046	0.563
Heart rate	−0.148	0.061
Body temperature	−0.010	0.904
Respiratory rate	−0.119	0.133
Pupil light reflex		
Both reactive	−0.292	<0.001**
One unreactive	0.008	0.923
Both unreactive	0.282	<0.001**
Pupil size (L)	0.357	<0.001**
Pupil size (R)	0.337	<0.001**

* *p* < 0.05; ** *p* < 0.005.

In the multiple logistic regression models, the adjusted odds ratio (OR) for predicting mortality was significant for age (OR 1.044, 95% CI 1.021–1.068; *p* < 0.001**) and left pupil size (OR 1.712, 95% CI 1.312–2.232; *p* < 0.001**) (Table 3).

**Table 3:** Multivariable regression analysis of factors independently associated with mortality.

Variable	OR	95% CI of OR	*p*
Age	1.044	1.021–1.068	<0.001**
Pupil size (L)	1.712	1.312–2.232	<0.001**

The overall mortality rate for patients with a Glasgow Coma Scale (GCS) score of 3 was 44.10%. Among these patients, those with a GCS score of 3 and both unreactive pupils with both pupils measuring ≥4 mm exhibited the highest mortality rate, which was 69.39% (34 out of 49 cases). In contrast, patients with a GCS score of 3 and both unreactive pupils with both pupils measuring <4 mm had the lowest mortality rate, at 32.26% (10 out of 31 cases). For a more comprehensive understanding of the association between pupil reactivity and size with mortality in patients with a GCS score of 3, the detailed information was provided in Table 4.

**Table 4:** The mortality rate in patients with combination of pupil reactivity and size for Glasgow coma scale scores 3.

	Total No	Mortality	Survival	Mortality rate
GCS 3 only	161	71	90	44.10% (71/161)
GCS3 and both reactive	52	12	40	23.08% (12/52)
GCS 3 and one pupil reactive	13	5	8	38.46% (5/13)
GCS 3 and both unreactive pupil	96	53	43	55.21% (53/96)
GCS 3 and both unreactive pupil, both pupil size<4 mm	31	10	21	32.26% (10/31)
GCS 3 and both unreactive pupil, one pupil ≧4 mm	15	9	6	60% (9/15)
GCS 3 and both unreactive pupil, both pupil ≧4 mm	49	34	15	69.39% (34/49)

In Tables 5–7, we observed varying results regarding the statistical significance of different variables concerning mortality and survival groups under distinct pupil conditions. Specifically, in Table 5, only the variable ‘age’ exhibited a statistically significant difference between the mortality and survival groups when considering individuals with both unreactive pupils and pupils with a size less than 4 mm. On the other hand, in Table 6, it revealed that ‘age’ was significantly different between these groups when one pupil had a size of 4 mm or greater. Lastly, Table 7 indicated that the variable ‘age’ showed significant differences between the mortality and survival groups when both pupils had a size of 4 mm or greater. However, the other variables did not display statistically significant differences in either case.

**Table 5:** Comparison of mortality and survival groups with both unreactive pupil and both pupil size <4 mm.

Variable	Overall *N* = 31	Mortality *N* = 10	Survival *N* = 21	*p*
Age, M (SD)	54.46 (18.04)	63.83 (13.85)	50.00 (18.36)	0.044*
BMI, M (SD)	23.50 (3.70)	24.48 (3.11)	23.03 (3.93)	0.318
Heart rate, M (SD)	93.87 (26.06)	91.70 (38.04)	94.90 (19.09)	0.755
Body temperature M (SD)	36.20 (0.82)	36.35 (1.33)	36.12 (0.45)	0.484
Respiratory rate M (SD)	16.81 (4.62)	15.80 (5.37)	17.29 (4.27)	0.411
Sex, *n* (%)				
Male	25 (80.6)	9 (90)	16 (76.2)	0.828
Female	6 (19.4)	1 (10)	5 (23.8)	
TTAS class, *n* (%)				
Level I	28 (90.3)	10 (100)	18 (85.7)	1.582
Level II	3 (9.7)	0 (0)	3 (14.3)	
Pupil size (L), M (SD)	2.16 (0.65)	2.30 (0.71)	2.10 (0.62)	0.422
Pupil size (R), M (SD)	2.18 (0.74)	2.20 (0.82)	2.17 (0.71)	0.909

**Table 6:** Comparison of mortality and survival groups with both unreactive pupil and one pupil ≧4 mm.

Variable	Overall *N* = 15	Mortality *N* = 9	Survival *N* = 6	*p*
Age, M (SD)	58.78 (20.56)	71.53 (8.41)	39.65 (18.39)	0.001**
BMI, M (SD)	23.60 (4.47)	23.70 (3.45)	23.45 (6.08)	0.921
Heart rate, M (SD)	84.8 (22.66)	76.67 (20.27)	97.00 (21.97)	0.088
Body temperature M (SD)	36.02 (0.25)	36.06 (0.21)	35.97 (0.32)	0.522
Respiratory rate M (SD)	20.53 (6.35)	19.56 (6.48)	22.00 (6.42)	0.485
Sex, *n* (%)				
Male	12 (80)	6 (66.7)	6 (100)	2.500
Female	3 (20)	3 (33.3)	0 (0)	
TTAS class, *n* (%)				
Level I	15 (100)	9 (100)	6 (100)	-
Level II	0 (0)	0 (0)	0 (0)	
Pupil size (L), M (SD)	3.63 (1.09)	3.44 (1.31)	3.92 (0.66)	0.433
Pupil size (R), M (SD)	3.47 (1.37)	3.56 (1.36)	3.33 (1.51)	0.771

**Table 7:** Comparison of mortality and survival groups with both unreactive pupil and both pupil size≧4 mm.

Variable	Overall *N* = 49	Mortality *N* = 34	Survival *N* = 15	*p*
Age, M (SD)	58.67 (16.68)	61.93 (15.20)	51.27 (18.02)	0.038*
BMI, M (SD)	24.17 (4.28)	24.32 (3.70)	23.81 (5.49)	0.704
Heart rate, M (SD)	82.63 (45.99)	83.71 (52.25)	81.20 (28.42)	0.809
Body temperature M (SD)	35.97 (0.40)	35.99 (0.38)	35.91 (0.46)	0.485
Respiratory rate M (SD)	13.65 (7.71)	12.94 (8.14)	15.27 (6.60)	0.336
Sex, *n* (%)				
Male	33 (67.3)	24 (70.6)	9 (60)	0.531
Female	16 (32.7)	10 (29.4)	6 (40)	
TTAS class, *n* (%)				
Level I	49 (100)	34 (100)	15 (100)	-
Level II	0 (0)	0 (0)	0 (0)	
Pupil size (L), M (SD)	4.96 (0.80)	5.00 (0.83)	4.87 (0.74)	0.594
Pupil size (R), M (SD)	4.98 (0.81)	4.97 (0.84)	5.00 (0.76)	0.908

**Figure 2:**
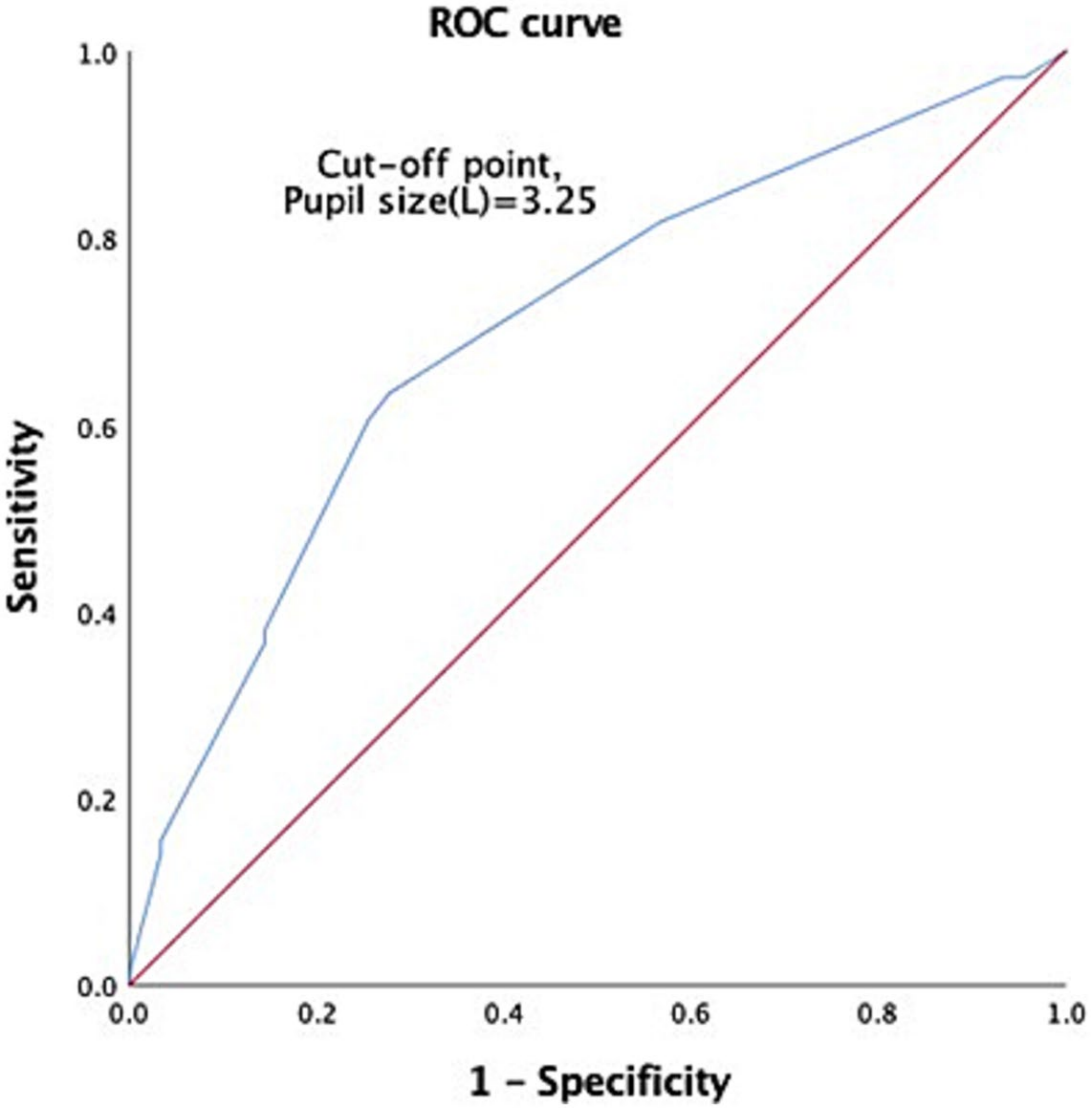
Receiver operating characteristics curve (ROC) to predict the probable mortality of TBI patients by measurement of age. A value of 41.11 y/o was considered the cutoff point, with AUC 0.689, 95% CI 0.608–0.770.

**Figure 3:**
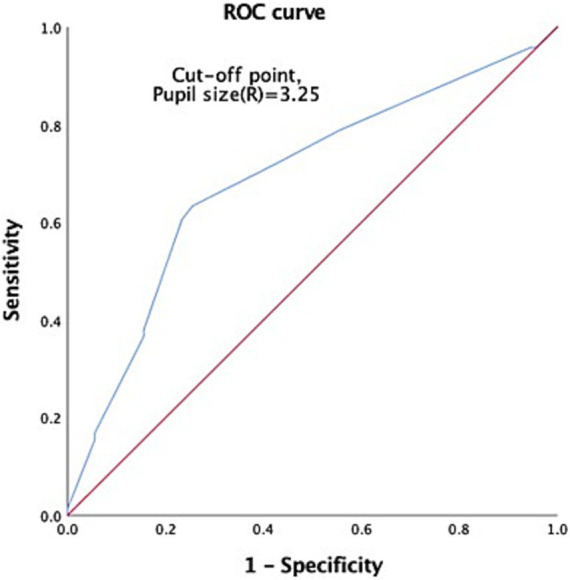
ROC to predict the probable mortality of TBI patients by measurement of left pupil size. A value of 3.25 mm was considered the cutoff point, with AUC 0.703, 95% CI 0.621–0.785.

**Figure 4:**
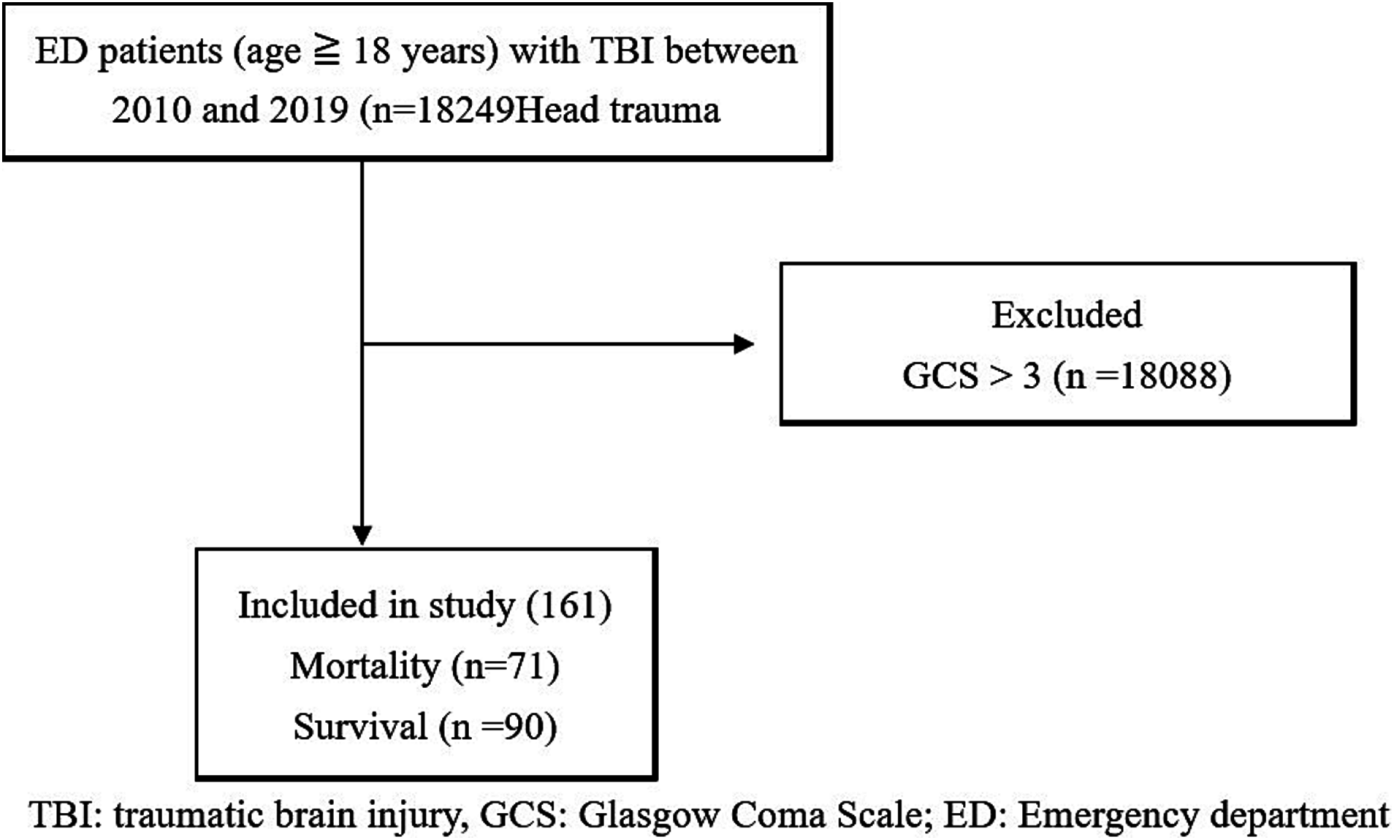
ROC to predict the probable mortality of TBI patients by measurement of right pupil size. A value of 3.25 mm was considered the cutoff point, with AUC 0.690, 95% CI 0.606–0.775.

Through ROC analysis and AUC calculations, we have identified key cutoff points in our study. For GCS 3 patients, we discovered an age cutoff point of 41.11 years, which strikes a balance between sensitivity (0.944) and specificity (0.367). The AUC for age was 0.689 with a 95% CI of 0.608–0.770 (Figure 2). Additionally, we established a reliable left pupil size cutoff point at approximately 3.25 mm, with an AUC of 0.703 (Figure 3). Furthermore, the cutoff point for right pupil size was 3.25 mm, and the AUC was 0.609 (Figure 4).

We also evaluated GCS 3 patients with unreactive pupils and those with GCS 3 plus unreactive pupils and both pupils ≥4 mm, taking into account age, left pupil size, and right pupil size AUC cutoff points, among other factors. This showed the cut off points for age and pupil size are 58.55 years and 3.75 mm (left), 3.25 mm (right), respectively in GCS 3 with unresponsive pupil light reflex. Detailed results are provided in Table 8.

**Table 8:** Cut off points for pupil size and age.

Category (numbers of patients)	Features	AUC	Cut-off point	95% CI interval	p	Sensitivity	Specificity	Youden index (MAX)
I. GCS3 (161)								
	Age	0.689	41.11	0.608–0.770	<0.001**	0.944	0.367	1.311
	Left pupil size	0.703	3.25	0.621–0.0785	<0.001**	0.634	0.722	1.356
	Right pupil size	0.609	3.25	0.606–0.775	<0.001**	0.634	0.744	1.378
II. GCS3+ unreactive pupil (95)								
	Age	0.736	58.55	0.634–0.838	<0.001**	0.679	0.714	1.393
	Left pupil size	0.668	3.75	0.558–0.778	0.005**	0.736	0.548	1.284
	Right pupil size	0.658	3.25	0.545–0.770	0.009**	0.755	0.571	1.326
III. GCS3+ unreactive pupil+ both pupil ≥4 mm (49)								
	Age	0.682	43.87	0.513–0.852	0.044*	0.941	0.4	1.341
	Left pupil size	0.540	5.25	0.367–0.713	0.657	0.294	0.8	1.094
	Right pupil size	0.482	6.5	0.482–0.655	0.845	0.029	1	1.029

Table 9 showed a comparison with related studies in TBI patients. Long study period (2010–2019) providing a comprehensive view. Our research includes a broader age distribution, encompassing older patients (21.2% are over 70 years old). Our study meticulously records both pupil size and reactivity. In contrast, some other studies either lack this data or do not provide corresponding information.

**Table 9:** A comparison with other related studies.

Study	Period	End-point	Mortality	Age distribution	Pupil evaluation	ICP monitor	Major findings
Kotwica et al. (9)	1985–1990	60 days Post-TBI	89% 99/111	18–82 yrs., < 50 yrs.: 77%, > 70 yrs.: 8%	No pupil data	No	All survivors were under 50 yrs
Lieberman et al. (29)	1999–2001	In hospital	FD 100% (104/104) NFD 33.3% (11/33)	Pupil fix dilate mean 40.6 yrs., Pupil not dilate mean 50.2 yrs	Presented with fixed/dilated pupils No pupil size recorded	No	Fixed/dilated: No survival chance; Non-fixed/dilated: aggressive treatment
Demetriades et al. (10)	1993–2002	In hospital	64% (320/497)	38 ± 18 yrs. (mean ± SD)	No pupil size or reactivity recorded	No	Age over 55: Significant mortality risk
Chamoun et al. (12)	1997–2007	6 months Post-TBI	49.2% (93/189)	36.5 (13–82) yrs. (mean (range))	Pupil dilate was defined as ≧4 mm But no pupil size recorded	Yes	Pupil size and reactivity key prognostic factors
Sadaka et al. (13)	2012–2016	6 months Post-TBI	80.6% (50/62)	No recorded	Pupil reaction No pupil size Recorded	Yes	6.9% with GCS 3 and bilateral fixed pupils achieved a good outcome
Current study 2023	2010–2019	In hospital	44.10% (71/161)	55.64 ± 17.41 yrs (mean ± SD) <50 yrs.:33.54%, 50–70 yrs.: 45.34%, > 70 yrs.: 21,2%	Recorded pupil reactivity and size	Yes	Odd Ratio in predicting the mortality was significant with age and left pupil size

## Discussion

### Novelty of current study

This study conducted a review of relevant literature and introduced an innovative approach to early mortality risk prediction in the emergency triage setting, without the need for brain CT scans or blood sampling. We offer a more precise method for assessing a patient’s risk by considering various variables, including the size and reactivity of the left and right pupils, which are combined to provide a comprehensive assessment. We have also established specific thresholds for age (58.55 years), left pupil size 3.75 mm and right pupil size 3.25 mm that are associated with predicting the risk of death in TBI patients with GCS 3 and unresponsive pupil light reflex. These distinctions can aid physicians in accurately evaluating the risk of TBI patients and in developing more effective treatment plans. Nonetheless, it is advisable for physicians to consider the results of multiple studies when making clinical decisions.

### Mortality consideration when combined GCS3 with pupil size and pupil light reaction

A GCS score of 3 at the initial assessment has been associated with high mortality rates: 49.2% (12), 64% (10), 80.6% (13), and 89% (9). In our current study, the mortality rate was 44.10%, similar to Chamoun RBs’ reported 49.2% and better than the others (Table 8). This discrepancy may be related to changes in healthcare practices (e.g., ICP-guided management), patient characteristics (such as age distribution), and the timing of the observation endpoint (whether in-hospital or 6 months later).

Our results (Table 4) suggest that when patients have a GCS score of 3 and both pupils are reactive, the mortality rate is 23.08% (12/52), similar to Chamoun RBs’ reported 23.5% (12). Additionally, we observed that patients with a GCS score of 3 and both unreactive and dilated pupils (≥4 mm) have a significantly higher mortality rate of 69.39%, which is lower than the 79.7% reported by Chamoun et al. (12). In contrast, patients with a GCS score of 3 and both unreactive pupils with both pupils measuring <4 mm have a lower mortality rate (32.26%) compared to the GCS of 3-only group (44.10%). These statistics indicate that the combination of a low GCS score and pupil reactivity has varying impacts on the likelihood of patient survival, and this information is crucial for understanding patient outcomes in cases of severe neurological impairment. Therefore, we want to emphasize the importance of measuring pupil size in predicting mortality.”

### Age is an independent risk factor to predict mortality

Numerous studies consistently underscore the pivotal role of age in predicting prognosis. For instance, in patients with a Glasgow Coma Scale (GCS) score of 3 suffering from traumatic brain injury (TBI), earlier research found that all survivors were under 50 years old (9), while individuals above 55 faced a notably higher mortality risk (10). In our current study, non-survivors had a mean age of 62.32 years, whereas survivors averaged 50.37 years (Table 1). This discrepancy may be attributed to the relatively advanced age of our patient cohort compared to that in previous studies (Table 8). It is plausible that older patients may have pre-existing chronic health issues that negatively impact their recovery ability (26), whereas younger individuals may possess more resilient brains better able to withstand damage (27). Additionally, treatment complications and effectiveness may vary between younger and older individuals, potentially influencing treatment outcomes (28).

We also reaffirm that age is an independent predictive factor for mortality (as shown in Tables 3, 5–7). Furthermore, we identified specific age thresholds of 41.11, 58.55, and 43.87 years in three categories with varying feature combinations (as presented in Table 8). In all three categories, age emerges as the most clinically significant feature, exhibiting the highest AUC and statistical significance. Notably, pupil size, especially right pupil size, holds less significance in Category III. These findings suggest that age outperforms pupil size as a predictor of patient condition in these categories. Importantly, age is a non-invasive and easily obtainable parameter, making it a practical clinical tool. The age threshold can be clinically applied to assist in decisions regarding TBI conditions.

### Left pupil size is an independent risk factor to predict mortality

Lieberman et al. reported 100% mortality in cases with fixed, dilated pupils (29). In the current study, both the size of the left (*r* = 0.357, *p* < 0.001**) and right (*r* = 0.357, *p* < 0.001**) pupils showed a significant correlation with mortality. This suggests that both left and right pupil size measurements appear to have potential clinical significance, and that patients with larger pupils have a higher risk of mortality (Table 2). Additionally, we observed that left pupil size had an odds ratio of 1.712 (*p* < 0.001**) for independently predicting mortality (Table 3). Furthermore, left pupil size, with a slightly higher area under the curve (AUC) value than the right pupil, was found to be a significant predictor of prognosis in different feature combinations (Tables 5–7).

The probable reasons for the left pupil size being a more meaningful determinant of prognosis compared to the right pupil size may relate to (1) left-sided dominance brain injuries could affect functions that are more closely linked to the left eye, which might be reflected in left pupil size, (2) specific injuries or conditions on the left side of the brain that we were not considered in this study.

In the current study, the setting was the triage of the ER, and no imaging studies had been conducted yet. Therefore, left hemisphere compression by hematomas, or cases with greater midline shift, were not considered as predictors. However, it’s possible that left pupil size was associated with more cases of left hemisphere compression by hematomas, or those with greater midline shift. In the future, it will be necessary to include the aforementioned parameters as predictors of mortality. Pupil size measurements can be valuable in assessing and stratifying the risk of TBI patients. Further research and clinical validation may be necessary to determine the most effective clinical applications.”

### Age and pupil as key predictors of mortality in TBI patients with GCS 3

Recently, several studies have shown that both age and pupil conditions are among the top predictors of in-hospital mortality after TBI (30–32). These studies also indicate that brain CT findings serve as important predictors. Our results are consistent with these findings. However, even without CT examination, we identified age and pupil size as key predictors of mortality in TBI patients with GCS 3 in the triage setting.

### Clinical implications

Our study underscores the significance of early mortality assessment in patients with a GCS score of 3, a group already recognized for its challenging prognosis. The recognition that age and pupil size serve as robust mortality predictors equips healthcare providers with valuable tools for making well-informed decisions in TBI patient management. This knowledge facilitates a more precise and individualized approach to care. For instance, patients exhibiting identified risk factors, such as advanced age or specific pupil size, can be closely monitored, and timely interventions can be initiated to enhance their chances of survival and minimize unfavorable outcomes while simultaneously improving their overall quality of life.

Furthermore, our study illuminates the necessity for additional research. While age and pupil size have emerged as prominent predictors, other variables have shown varying degrees of significance. This highlights the potential for further investigations into additional factors that might contribute to TBI patient outcomes, thus leading to a more comprehensive understanding of prognosis and the development of more effective treatment strategies.

### Strength and limitations

Our study possesses several strengths. Firstly, the data in the current study is the most recent, covering the period from 2010 to 2019, thus reflecting the latest advancements in modern medical practices and technologies. Secondly, unlike other studies, our research includes a broader age distribution, encompassing older patients (22% over 70 years), which enhances our ability to gain a more comprehensive understanding of prognosis across various age groups. Thirdly, our study meticulously records both pupil size and reactivity, both of which are critical prognostic factors. In contrast, some other studies either lack this data or do not provide corresponding information.

However, it’s important to acknowledge several limitations in our study. Firstly, as a retrospective observational study, there is the possibility of miscoded feature variables. Researchers have limited control over the data collection process, which may introduce biases or confounding factors. Secondly, in comparison to some other studies, the current study has a relatively smaller sample size (161 cases), potentially constraining its statistical power and limiting the generalizability of findings to larger and more diverse patient populations. Thirdly, the accuracy of GCS scores and the consistency in measuring pupil size and reactivity may vary among different healthcare providers, potentially leading to measurement errors. Fourthly, the study, conducted at the ER triage, did not account for the existence of uncontrolled or unmeasured confounding factors that could influence the results. For instance, other medical conditions, various treatments, surgical procedures, and complications that could impact the outcome after TBI were not considered in the analysis. Finally, variations in study endpoints, with some studies focusing on in-hospital outcomes while others use a 60-day endpoint, may affect the comparability of study results.

Consequently, there is a need for larger prospective studies with more comprehensive data collection and the inclusion of additional variables to be considered in future research.

## Conclusion

In conclusion, our research highlights the practical significance of age and pupil size as key predictors of mortality in TBI patients with a GCS score of 3. These findings offer valuable insights for healthcare providers and researchers, enabling them to make more informed decisions in the assessment and management of TBI patients. By taking these factors into account, we can improve patient prognosis and increase their chances of survival.

## Data Availability

The raw data supporting the conclusions of this article will be made available by the authors, without undue reservation.
